# Accelerating Reaction Network Explorations with Automated Reaction Template Extraction and Application

**DOI:** 10.1021/acs.jcim.3c00102

**Published:** 2023-05-22

**Authors:** Jan P. Unsleber

**Affiliations:** † Laboratory of Physical Chemistry, ETH Zurich, Vladimir-Prelog-Weg 2, 8093 Zurich, Switzerland

## Abstract

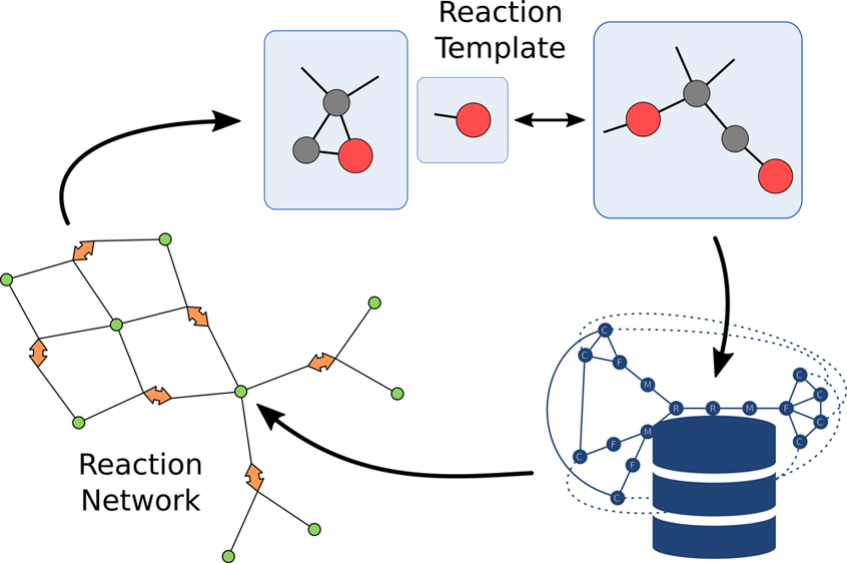

Autonomously exploring chemical reaction networks with first-principles methods can generate vast data. Especially autonomous explorations without tight constraints risk getting trapped in regions of reaction networks that are not of interest. In many cases, these regions of the networks are only exited once fully searched. Consequently, the required human time for analysis and computer time for data generation can make these investigations unfeasible. Here, we show how simple reaction templates can facilitate the transfer of chemical knowledge from expert input or existing data into new explorations. This process significantly accelerates reaction network explorations and improves cost-effectiveness. We discuss the definition of the reaction templates and their generation based on molecular graphs. The resulting simple filtering mechanism for autonomous reaction network investigations is exemplified with a polymerization reaction.

## 1 Introduction

Due to advances in computer hardware and quantum chemical methods, it has become possible to model and investigate chemical processes with atomistic resolution at an unprecedented scale. A key concept in this field of research is the concept of chemical reaction networks, which are networks of compounds and their reactions, encoding the chemical transformations that underlie a given chemical process. With the aforementioned advances in computational chemistry, the large-scale automated exploration of chemical reaction networks has been a new and growing research area in the past years. For recent reviews, see refs (1−7).

A key advantage of such automated explorations based on first-principles electronic structure methods is that they can be bias-free. However, these exploration campaigns can still quickly become unfeasible due to the complex nature of the chemical reaction networks. Generally, any compound in the reaction network can react with any other compound and generate at least one new compound. The new compound can then again react with all existing ones. It is thus clear to see that unconstrained explorations of arbitrary reactions lead to a combinatorial catastrophe.^7^ Even explorations with some level of guidance may yet explore regions of the reaction network that are of no interest to the operator. In order to avoid these pitfalls in the exploration of reaction networks, it is possible to have close supervision of the exploration process by an operator. The operator would need to deem any compound and reaction in the network as “of interest” or ’unimportant’. However, this possibly introduces back the operator’s biases, and for more extensive explorations, this method of steering or filtering the exploration is too time-consuming to be feasible.

As a result, more automated ways of steering and filtering are required. Here two orthogonal possibilities arise from the structure of the reaction networks. These two ways of steering can, of course, be combined. First, it is possible to restrict the viable compounds for continued reaction trials. While operator-generated filters may be applied, a more natural way of filtering compounds is the analysis of microkinetic models of the existing reactions.^8,9^ Thresholds based on the concentrations or flux calculated from the time propagation of given starting conditions can be applied to filter the compounds picked for further reaction trials. While this reduces the combinatorial problem of compound combinations, this filtering does not affect the possible combinatorial explosion of potential reactions with growing compound sizes. Hence, second, it is possible to restrict the reactions explored for a given set of compounds. Again it is possible to generate simple hand-crafted rules akin to “molecules with nonzero charges of the same sign may not react”. However, more automated filters have been proposed in previous works. One ansatz is heuristics based on *in situ*, *ab initio* calculations of molecular properties.^10,11^ These approaches are based on accurate local information about singular compounds and aim to transfer it to the reactions. They extrapolate from isolated compound properties to reactivities of compound pairs.^11^ These approaches have the benefit that required compound properties are available in linear time with respect to the number of compounds in the network. Still, they have the inherent problem of using low-energy structures to guess the properties of high(er) energy transitions and structures (transition states).^11^ A common set of theses compound-based descriptors is derived from conceptual density functional theory (DFT).^12−17^

**Figure 1:**
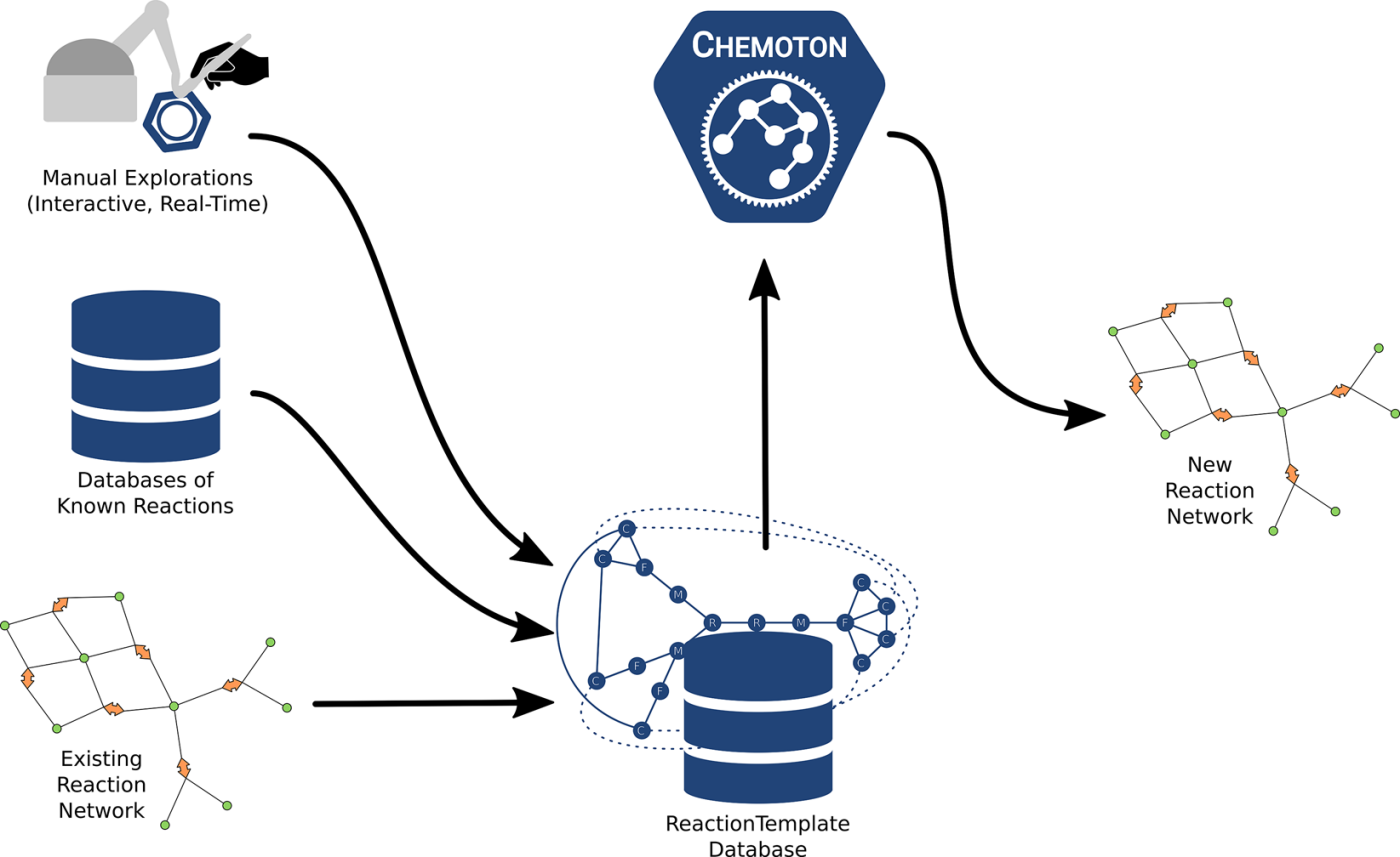
General workflow of a reaction network exploration including filtering by reaction templates with Chemoton.

In contrast to compound-based data and heuristics, this work will provide a simple method with which knowledge about existing reactions can be encoded, abstracted, and subsequently used as a filter to explore new reactions. This method is the extraction and application of reaction templates that encode reactions. Hence, the reaction-centered templates have the opposite working mechanism of compound-based heuristics. They store reaction-centered data and extrapolate between compounds, while compound-based heuristics encode compound-based data and extrapolate toward the possible reactions. The assumption made with reaction templates is that similar compounds will likely allow the same reactions to occur. Here, the exact definition of the reaction templates then defines what we mean by “similar compounds”. A downside of the reaction templates is that they require prior knowledge of possible reactions. This prior knowledge can possibly be collected from multiple different sources. The general workflow is shown in Figure 1 and collects these sources of knowledge into three main groups that will be discussed in a later section.

The Article is structured as follows: first, the chosen reaction template definition and possible alternatives are introduced and discussed, and afterward, their generation, deduplication, and usage are discussed. Subsequently, the polymerization of ethylene oxide is investigated, highlighting the efficacy of the generated reaction templates to accelerate the autonomous exploration of reaction networks. The work closes with a short discussion of future work and conclusions.

## 2 Theoretical Background

In this work, molecules are described as molecular graphs consisting of atoms (nodes) connected with bonds (edges). The specific graph representation defined by Sobez and Reiher^18^ (available in the program Molassembler(19)) annotates atoms with additional information, such as the element type and the local shape of its coordination sphere. Similarly, bonds are annotated with information that encodes the rotational freedom around this bond. If the rotation is hindered, these annotations track the relative alignment of the coordination shapes positioned on the atoms at each end of the bond. Based on this definition of molecular graphs, we can define reaction templates that encode changes to these molecular graphs. In the following sections, we will describe how common graph-based algorithms can be utilized to define, compare, and apply reaction templates such that they are effective filters in reaction network explorations.

### Reaction Template Definition

The reaction templates aim to abstract multiple similar reactions into a single template in the simplest way possible. At the same time, they shall be specific enough such that reactions that are somewhat different are part of a different template. In essence, each template shall encode meaningfully different chemistry while keeping the number of required templates as small as possible. Generating templates and their usage must be feasible, avoiding graph manipulations with unfavorable time complexity. Additionally, template definitions that are easily interpreted and visualized, thus helping to explain the underlying chemistry to an operator, are preferable. Given that the goals and requirements above are somewhat abstract, we illustrate them by quickly discussing counterexamples of template definitions that fail to adhere to them. This will lead to the final definition of the template and motivate why it was chosen.

**Figure 2:**
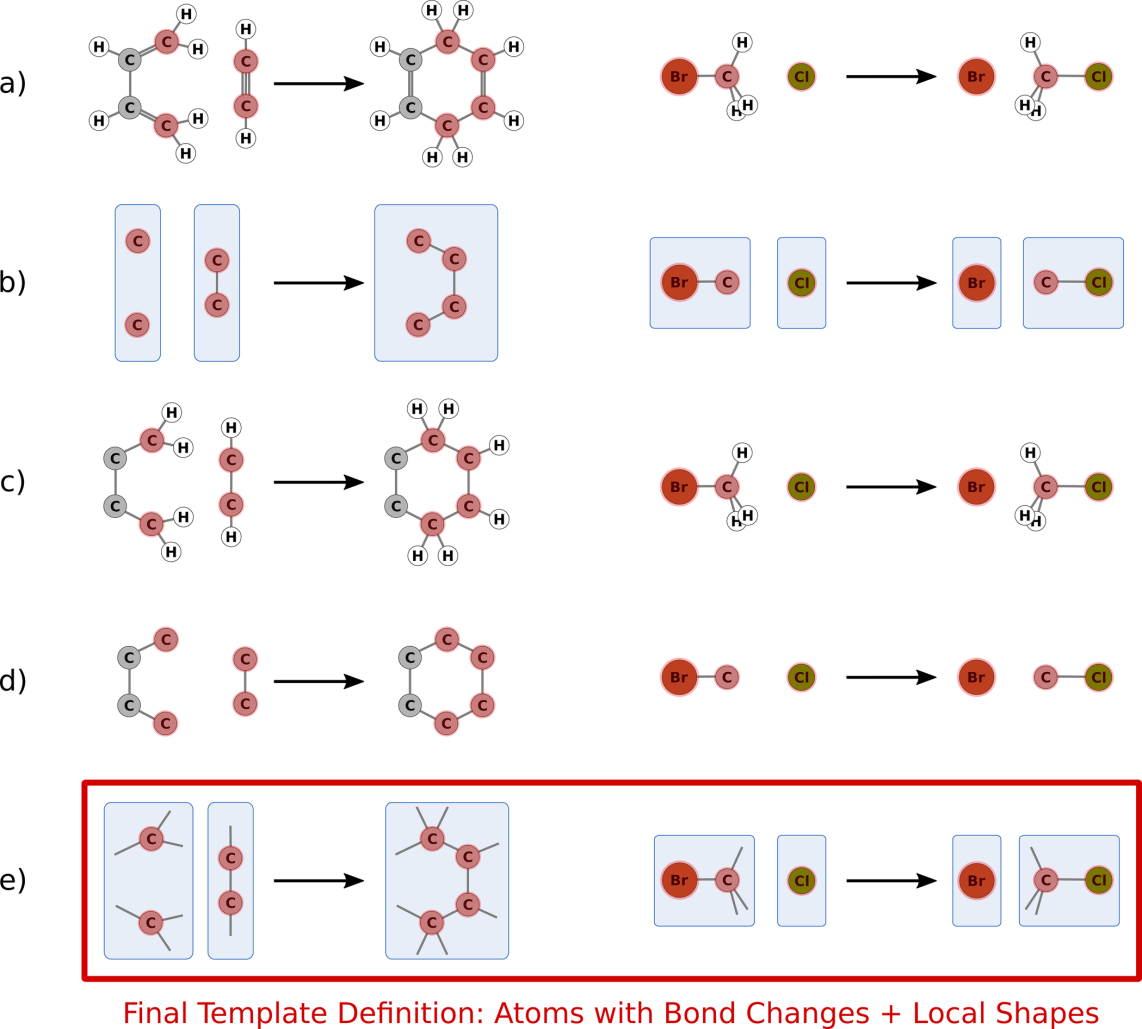
Two example reactions: on the left, a Diels–Alder reaction, and on the right, a prototypical S_N_2 reaction.

Figure 2a shows two reactions in which the atoms that change their bonding pattern are highlighted in red. Given that it is not straightforward to determine the exact bond orders in molecules *ab initio*, we will consider bonds as binary, avoiding technical difficulties and restricting graph analyses to the existence of edges rather than their weights. As a result, not all carbon atoms in the diene are highlighted.

We would like to note that it would also be possible to describe this approach as tracking additional information about the columns and rows in the so-called bond and electron (BE) matrix.^20^ The counting of elements in such a BE matrix is similar to how some automated reaction exploration software packages restrict explorations.^21−23^

Figure 2b shows the most simple template resulting from bookkeeping the atoms with changing bonding patterns and the fragments and molecules they are in. It can be easily seen that any two carbon atoms in the same molecule would now be identified as what should be the diene of the Diels–Alder reaction. This template definition is thus not specific enough to separate different reactions.

A simple expansion would be to track the nearest neighbors of atoms with changing bonding patterns too.^24,25^ Examples of this template type are shown in Figure 2c. Here, we can see that the dienophile of the Diels–Alder reaction is now too tightly defined. Any dienophile that is not acetylene would be excluded.

**Figure 3:**
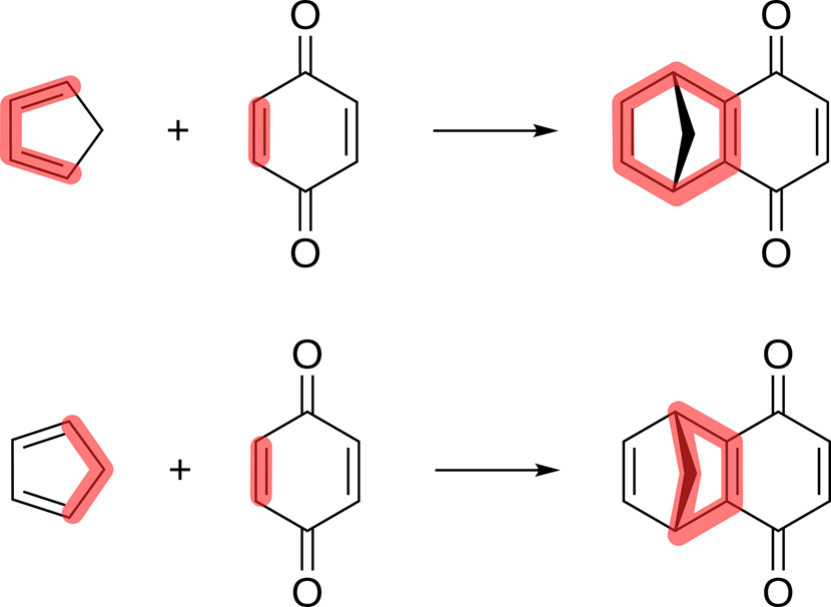
Example of the Diels–Alder reaction with a cyclic diene. Top: the expected template fragments based on chemical intuition. Bottom: the resulting template fragments when employing a minimum spanning tree of reactive atoms per molecule.

A more involved template definition would require tracking of exactly one single fragment per molecule. This particular possibility is shown in Figure 2d. While the generated template at first glance strikes a decent balance in its specificity to the molecular structure, it is expensive to compute the (minimal) spanning trees required for this template definition. Additionally, the same fragment that encodes the chemical intuition may not be the minimal spanning tree but another more complex one that only becomes apparent when multiple reactions are compared. For example, we can look at the Diels–Alder reaction of cyclic dienes (see Figure 3).

This work’s chosen reaction template definition aims to avoid these pitfalls. It still relies on the fragments defined in the most minimal ansatz (see Figure 2b) but also dresses each node with additional information about the local coordination shape. This local shape information includes information akin to the hybridization concept and is readily available for molecules described as Molassembler graphs. The local shapes are indicated by dangling bonds in Figure 2e. The final template definition can be summarized as follows.

All atoms with changing bonding patterns are included. Atoms are identified by their element type. Atoms that are included and connected are grouped into fragments. Fragments are grouped by molecule. For each atom, a list of possible shapes is stored. Hence, a single template can include multiple allowed local shape combinations.

### Reaction Template Generation

All algorithms required to build and apply the reaction templates defined in this work are implemented in the program in Art. The minimal requirements for generating a single reaction template are the molecular graphs for all starting materials and product compounds of a given reaction and an exact mapping of all atoms from one side of the reaction to the other. In practice, Molassembler, the program implementing the basic algorithms for molecular graphs, can generate graphs from atomistic 3D structure data (such as “.xyz” files) and bond information (e.g., bond orders from electronic structure calculations). Optionally, it is possible to store known reaction barriers for a given template. This information about thermodynamics can be used to filter which templates are applied in subsequent reaction network explorations.

Three main data sources for the generation of reaction templates in practical applications can be identified and are represented in Figure 1:

1. Manual creation of reaction templates by an operator. Each template is constructed from at least one handcrafted example reaction.
2. Automated extraction of reaction templates from existing reaction networks by analysis of stored reactions and elementary steps.
3. Translation of databases of known reactions or predicted reactions into the described template format.

We will briefly comment on each of them and the status of their implementation.

1. Generally, any molecular builder can create two sets of structures considered the end points of a reaction, and subsequently, these can be analyzed for the corresponding template. Similarly, two snapshots of a molecular dynamics trajectory can be analyzed. In both cases, the operator must ensure the correct atom mapping. An option to generate reaction templates in this fashion has been added to SCINE Heron and will be made publicly available in the near future. This integration in SCINE Heron also allows generating trajectories of reactions with real-time haptic feedback;^26−30^ the reactions explored in this interactive manner can also be analyzed.
2. The automated analysis of the existing reaction networks stored in a SCINE Database is a core feature of SCINE Art and is available in version 1.0.0.
3. Importing from other databases is possible if the data required for molecular graphs or 3D structure examples with atom mapping can be read. The translation of reaction suggestions based on machine-learning techniques is being investigated but, depending on the data representation, suffers from the missing, exact mapping of all atoms. We have recently discussed similar problems in the context of the automated validation of retrosynthesis suggestions.^31^

### Template Deduplication

**Figure 4:**
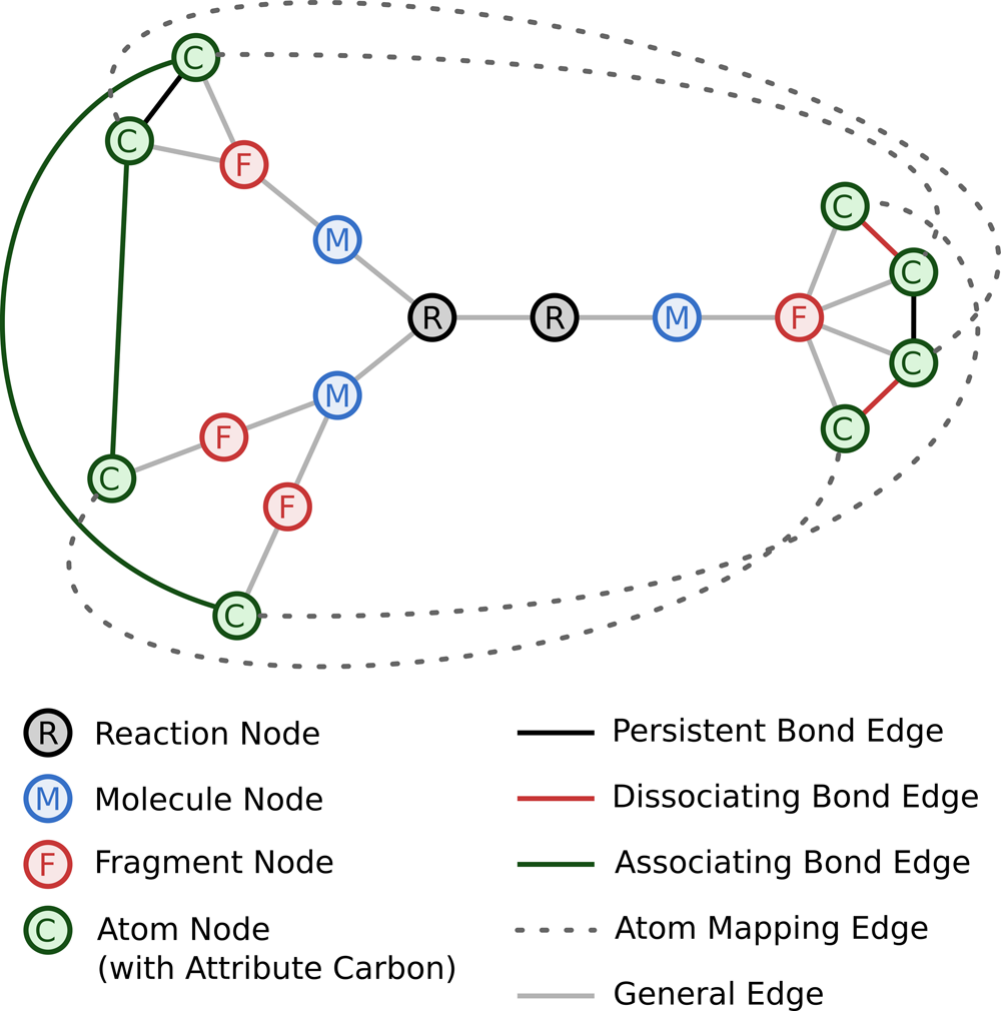
Example graph for an entire reaction template as generated in the deduplication of reaction templates. The full Diels–Alder reaction encoded by the graph is shown in Figure 2.

To use the reaction templates in automated reaction network explorations, it is essential to store, compare, and deduplicate the templates. It is possible to deduplicate the templates by comparing a combined reaction template graph. One such graph, for Diels–Alder reactions, is shown in Figure 4. Comparisons, checking if two reaction templates describe the same reaction, are then carried out as exact graph isomorphism checks of the corresponding reaction template graphs. The graph describes atom mapping from one side of the reaction to the other (dashed gray lines) and the relation of atoms within one side into fragments and molecules. Furthermore, the graph encodes which bonds are broken or formed during the templated reaction. Based on the flavor of the reaction template, atom nodes may be dressed with additional information, such as local shapes or nearest neighbor atom types. Removing the fragment nodes from the graph would be possible, as their information is also stored in the bond edges (solid black, red, or green edges). However, they are kept for visualization purposes and possibly faster terminations of graph comparisons in cases where reaction template graphs do not match.

Comparing the template graphs shown above may allow for additional higher-level analyses of the reactions occurring within a reaction network or comparisons between two networks. Standard metrics such as the graph edit distance (GED)^32^ between two template graphs, but also kernel-based comparisons, are possible candidates.

### Accelerating Reaction Network Explorations

**Figure 5:**
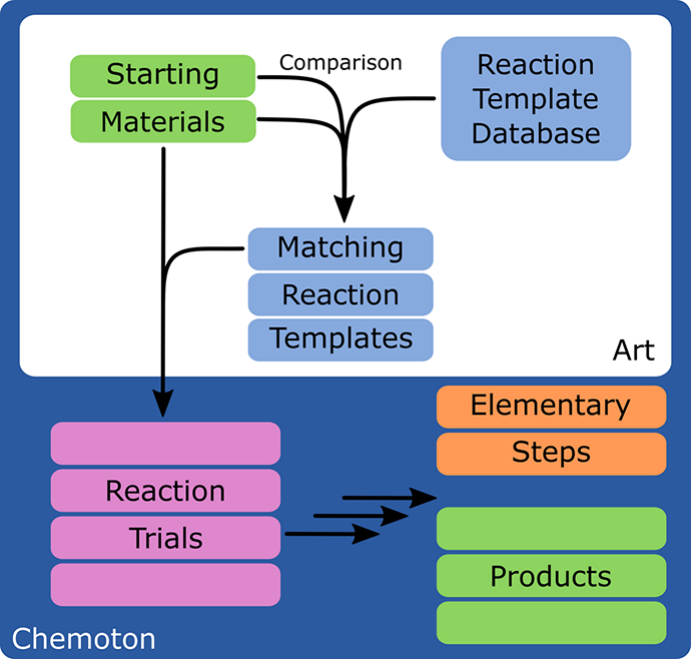
Workflow generating new elementary steps and structures based on reaction templates and existing structures, as implemented in SCINE Art and SCINE Chemoton.

As discussed in the Introduction, reaction templates accelerate the exploration of reaction networks by filtering the number of trialed reactions. Any compound or compound combination within the network to be probed for its reactions is first checked against the existing reaction templates. The templates that match the compound combination are gathered, and only those reaction trials which aim to generate a reaction encoded within these templates are performed. As implemented in SCINE Chemoton, this workflow is depicted in Figure 5. In order to test if a template matches a set of reagents, the molecular fragments that define the template are searched for in the given reagents’ molecular graphs. This identification is carried out with a standard subgraph matching algorithm. If all molecular fragments encoded in either side of the template can be identified without overlapping atoms, the template is applicable to the given reactants. It is possible that a single template can be applied in many different ways to a given set of reactants. If this is the case, all possibilities are reported. It is then possible to extract the expected bond changes or to generate the product graphs by application of these bond modifications to the molecular graphs of the reagents. As a result, there are at least two options for the generation of the allowed reaction trials:

1. Generating the product graph(s), by application of the template to the starting material(s). Then it is possible to generate a 3D representation of the products, find a proper alignment of reactants and products, and run any double-ended elementary step search algorithm (such as CI-NEB^33^).
2. Analyzing either the product graph or the information within the template directly, it is possible to bias a single-ended elementary step search algorithm (such as AFIR,^34^ GSM,^35^ or NT2^23^) toward the intended product.

The first option has the clear advantage that it should find the best elementary step in a single algorithm run. A downside is, in practice, that double-ended methods often require a more involved alignment procedure for reactants on both sides of the reaction. The second option may likely require multiple single-ended runs per applied template, and albeit for advanced algorithms, the recovery of the reaction may not be guaranteed. However, its advantages are that the alignment of the reagents is simpler and that multiple runs of the algorithms may lead to the serendipitous finding of additional important reactivity, assuming that templates (by proxy) encode reactive sites in the molecules. It is to be expected that the second option is more computationally demanding.

In this work, we will focus on the second option employing the NT2 algorithm, for three reasons:

1. As we have described previously,^23^ the NT2 algorithm is constructed so that it does not enforce given bond rearrangements but only biases toward them. Hence, it is interesting if it can be applied sufficiently well in this context, where a particular rearrangement is intended.
2. This exact algorithm will also be used to generate a reference network from which reaction templates are extracted. Therefore, a one-to-one comparison of cost and results with and without relying on templates is possible.
3. The alignment procedure required for double-ended searches, based on the graphs generated by the template application, is not yet fully developed in the current framework.

## 3 Computational Methodology

### Exploration Setup

All quantum chemical explorations were carried out with Puffin 1.1.0^36^ and a modified version of Chemoton 2.2.0.^23,37^ These modifications to Chemoton are part of the release of Chemoton version 3.0.0. All reactive trials were carried out with the NT2 algorithm.^23^

The quantum chemical raw data (e.g., electronic energies and nuclear gradients) were obtained with GFN2-xTB.^38,39^ All calculations were done in the restricted open-shell formalism and the *C*_1_ point group symmetry. All calculations included implicitly modeled solvent, tetrahydrofuran (THF), described with the generalized Born model with surface area contributions (GBSA) as implemented in Xtb v6.5.1.^40,41^ Timings were recorded on AMD EPYC 7742 processors.

The key settings of the reaction network explorations, mainly energy and molecular mass-based thresholds limiting the exploration space, are discussed in the sections below. Additional, nondefault settings for exploring the reaction networks are summarized in the Supporting Infomation.

## 4 Application to Polymerization Networks

**Figure 6:**
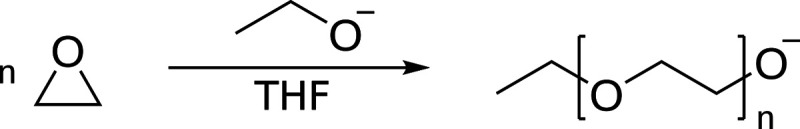
Anionic polymerization of ethylene oxide.

To investigate the performance of the template definition given above, we investigate the anionic polymerization of ethylene oxide; see Figure 6. The exploration of the polymerization process is split into two phases. First is the template generation phase. A short yet exhaustive, template-free exploration of only the starting materials is conducted, constrained to a low molecular mass in this initial phase. The resulting network is analyzed, and templates are extracted. A single template is also generated by hand using a prerelease of SCINE Heron and its real-time quantum chemistry molecular viewer. In the second phase, the template(s) extracted from the initial network exploration or the manual generation are applied in an exploration that again starts from the starting materials but allows explorations toward larger molecular masses.

Applying reaction templates to polymerization reactions may seem like an obvious choice, given that polymerization usually progresses by a constant pattern of reactions. However, it is, at the same time, a challenging example because a template that matches any part of the polymer will also match all repetitions of the said substructure. Furthermore, the example is limited to three atom types, and any serendipitous side reaction found with the NT2 protocol will likely lead to additional species that match additional templates. For this reason, the effect of templates on the amount of computational work required can be assessed especially well in this example.

### Initial Template Generation

In the initial template generation, ethylene oxide and the ethoxide anion were added into a reaction network as starting materials. Subsequently, an exploration based on GFN2-xTB (implicit solvent: THF, see Section 3) was carried out. The exploration was limited to bimolecular reactions of ethylene oxide with ethoxide, bimolecular reactions of two molecules of ethylene oxide, and unimolecular transformations of ethylene oxide and ethoxide. Essentially, this comprises all unimolecular reactions and all bimolecular combinations where the two molecules do not present an overall charge with the same sign. This initial exploration was configured to exhaustively explore the network allowed within the boundaries described. The exploration is essentially restricted to the molecular mass of the product with *n* = 1. The exact settings are given in Table S1 in the Supporting Information. Exhaustive refers to the reaction trials generated per reactant structure or reactant structure combination. Only one conformer per reactant was screened for elementary steps to simplify and speed up the exploration. No explicit conformer sampling was carried out. Additionally, two thresholds for Δ*G*^‡^ and Δ*G* are introduced as constraints on the kinetics and thermodynamics of the explored network. All compounds reachable from active compounds by at least one reaction with a barrier lower (Δ*G*^‡^) than 80 kJ/mol and a reaction energy (Δ*G*) lower than 50 kJ/mol are considered active. This analysis is initialized by setting the two starting compounds as active. In general, inactive compounds will not be considered for further reactions by Chemoton. The resulting network was analyzed, and all reactions were translated into templates. Subsequently, the templates were joined or deduplicated as described previously.

This process mimics the use case where no prior knowledge of a given process exists, and templates have to be generated by an exhaustive exploration first. The key is then that the initial exploration is not limited in the reactivity but limited to a small set of representative compounds representing all future compounds as best as possible. Another instance of such an investigation would be catalytic systems of transition metal complexes where innocent ligands are screened. An initial exploration of a representative set of ligands could generate reused templates for accelerated larger screening campaigns.

**Figure 7:**
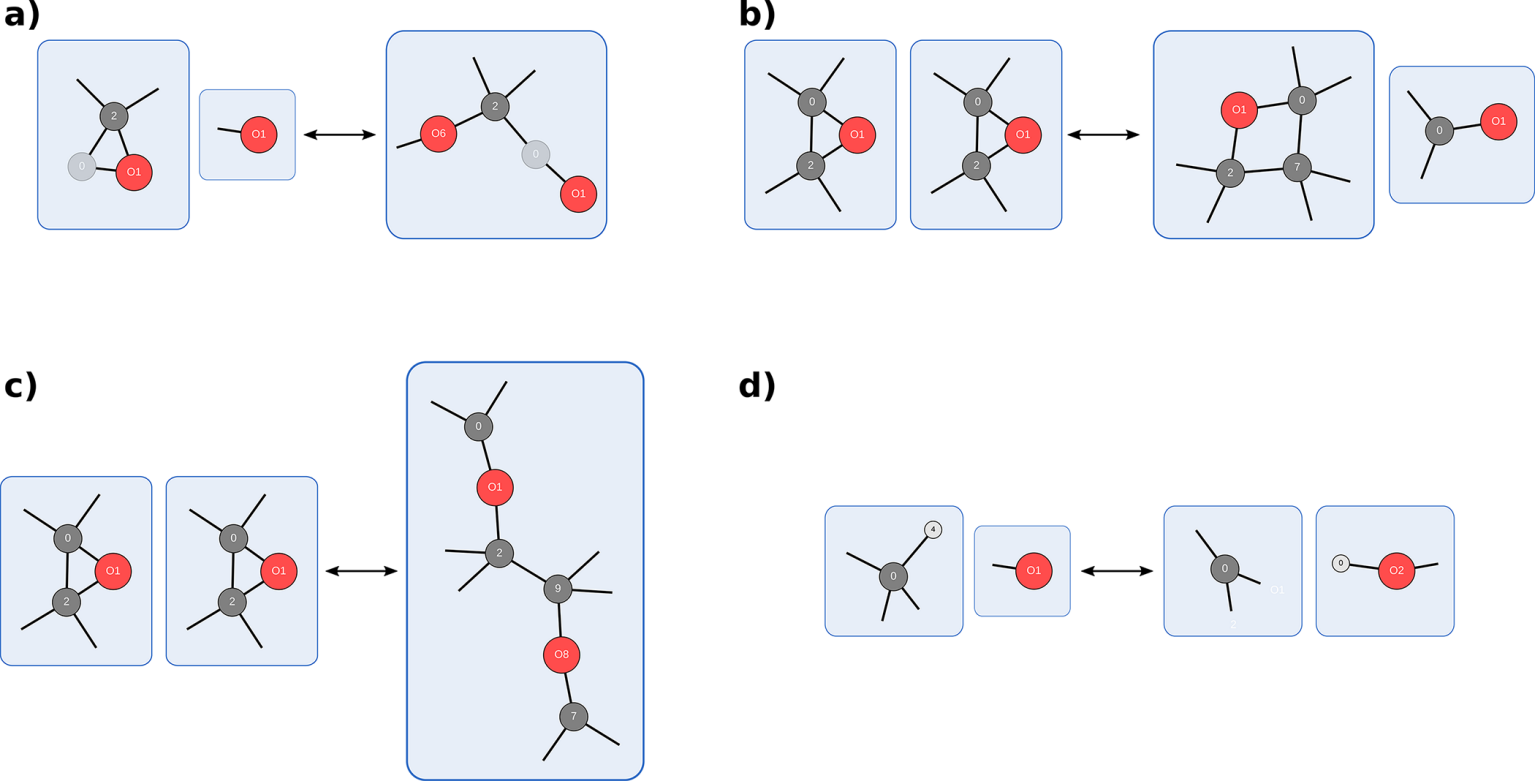
Example reaction templates. The local shape of each atom is indicated by dangling bonds. Blue boxes indicate the molecule boundaries. The two opaque carbon atoms in example (a) are not part of the template but are added to visualize that this template matches the chain elongation in the anionic ethylene oxide polymerization.

In this investigation of a polymerization process, the database of all templates extracted from this initial reaction network includes 79 unique templates, and five compounds (including the two starting compounds) are finally labeled as active. An excerpt of the templates is shown in Figure 7. Additional tests with other unpublished reaction networks show that several hundreds of templates can be extracted in larger, more diverse reaction networks. In many cases, a large portion of the templates encode reactions that are not relevant at ambient conditions, which is why it is possible to limit the template extraction process to reactions that have low barriers (below a user-defined threshold). Nonetheless, it is expected that databases with multiple thousands of templates will result from larger translation campaigns based on existing data sets or accumulation across multiple reaction networks.

The newly discovered three active compounds result from two reactions. One reaction is the expected first polymerization step. The other is a hydride abstraction from the ethanolate yielding acetaldehyde.

**Figure 8:**
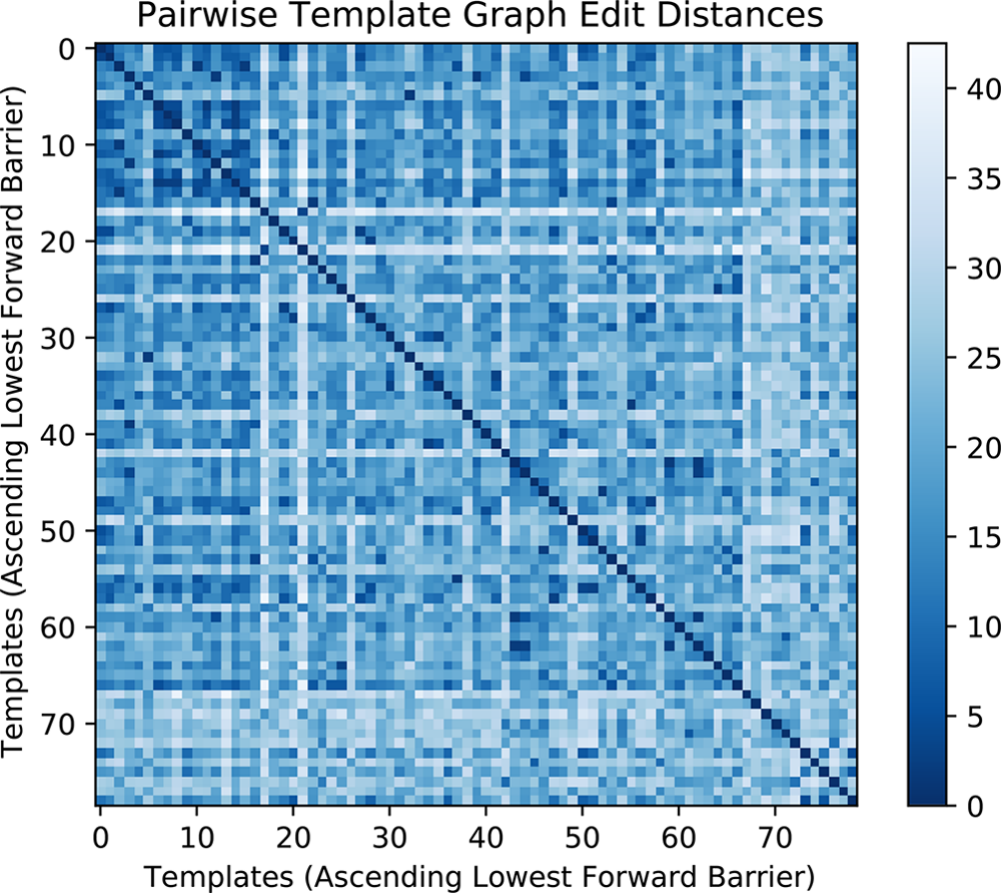
Pairwise graph edit distances (GEDs) of the initial 79 reaction templates recorded for the anionic ethylene oxide polymerization.

A simple analysis of the template graphs shows that, on average, those templates with a lower forward reaction barrier recorded are more similar to one another than those with larger barriers tallied. This is visualized in Figure 8, where the similarity metric for template graphs is the pairwise graph edit distance (GED). A simple explanation may be that the same bonds and atoms are involved in the reactions with lower barriers.

In addition to the automated extraction of reaction templates, the main template for the polymerization process (Figure 7a) was also generated with the graphical user interface Heron. The resulting second database of exactly one template mimics the application where an operator decides on a predetermined set of allowed reaction templates. This use case is akin to the workflow of many common computational investigations of chemical systems based on expert guesses. The following section will report how the two reaction template databases (79 automatically extracted templates and one manually generated template) are applied to accelerate explorations limited to higher molecular masses of the polymer.

### Automated Exploration with Reaction Templates

The sets of templates generated by the procedures described in the previous section are tested throughout this and the following sections. For the application of the reaction templates, an additional energy threshold exists. This threshold determines whether a matching template generates an elementary step trial for given reactants. In the reaction direction determined by the reactant matching, the lowest recorded barrier of a given template is compared against the threshold. If the recorded barrier is lower than the threshold, the reaction encoded by the template is probed, and elementary step trials are generated. Unless stated otherwise, all templates that fit a given set of reactants and have a recorded barrier lower than 90 kJ/mol are accepted and applied in the following explorations. Assuming that the reaction barriers tallied for the templates may change when applied to new structures, the template application threshold was chosen higher than the threshold set for the categorization of compounds to be active (reachable with a barrier Δ*G*^‡^ < 80 kJ/mol). This chosen threshold allows 25 of the 79 templates to be applied if the reactants match the template.

To validate the reaction templates, we first repeated the exact exploration that yielded the reaction templates, now, however, applying the templates rather than carrying out a brute-force exploration. The same compound filters and restrictions of reaction barrier and reaction energies for compounds’ active/inactive categorization were applied. The results of these explorations are summarized in row 3 of Table 1. It can be seen that, as expected, the template-based run requires significantly fewer computational resources compared to the initial exploration (Table 1, row 1). Similarly to the CPU time, the number of reaction trials and the number of compounds found in total also decrease when increasing the restrictiveness with the templates. Furthermore, it can be seen that the success ratio of the trials allowed by the templates is much higher than that of all brute-force trials. (The success ratio of elementary step trials is defined as the number of elementary step trials that yielded elementary steps divided by the total number of trials.) A manual inspection of the 10 resulting compounds of the template-based exploration of PEO showed that all compounds deemed active according to the simple thresholds of reaction barriers and reaction energies are recovered.

**Table 1:** Condensed Results for Reaction Network Explorations of the Polymerization Reactions for Poly(ethylene oxide) (PEO) and Poly(propylene oxide) (PPO), with Varying Sets of Templates and Mass-Determined End Points

exploration				reaction trials		network statistics	
no.	reaction	template	end point	count	success ratio	compounds	CPU·time
1	PEO	No	*n* = 1	52 490	23.7%	90	557 h
2	PEO	No	*n* = 2	≫25 000 000			≫40a
3	PEO	Yes (25)	*n* = 1	166	47.6%	10	2 h
4	PEO	Yes (25)	*n* = 2	6 415	30.8%	479	72 h
5	PEO	Yes (25)	*n* = 3	>15 000 000		>50 000	>25a
6	PEO	Yes (1)	*n* = 20	378	57.1%	22	936 h
7	PPO	Yes (25)	*n* = 1	181	43.1%	28	2 h
8	PPO	Yes (25)	*n* = 2	1355	31.8%	155	32 h
9	PPO	Yes (1)	*n* = 5	640	71.6%	124	68 h

Given that the re-exploration of only five active compounds stemming from two reactions is not a significant statistic, additional tests with allowed barriers in the explorations and for the template application of 90 and 100 kJ/mol, respectively, as well as 100 and 150 kJ/mol, were carried out. All of the reactions and compounds were recovered in these cases as well. However, in the case of 100 and 150 kJ/mol thresholds, one reaction would not have been included in the active part of the reaction network, as the template run found only reaction channels with barriers of 102 kJ/mol and greater. In contrast, the exhaustive initial exploration found additional channels with a 94 kJ/mol barrier. It stands to reason that a slightly higher threshold for the template-based exploration than the initial exhaustive exploration would be a reasonable remedy for this shortcoming. Applying reaction templates will result in fewer reaction channels found per reaction as fewer elementary trials are run. If the elementary step trials are run with the exact same algorithm and settings in both the brute force and the template case, the channels found in the template case will be a subset of those in the brute force case. The minimal barrier channel does not necessarily have to be part of this subset. An alternative remedy would be to adjust the exploration settings for the template case such that each template reaction is probed more exhaustively than in the brute force case, reducing the chance of missing a relevant channel.

Continuing the explorations of the generated reaction networks, converging them to a higher molecular mass limit is possible. This limit was chosen to be equivalent to a product with *n* = 2 in the reaction shown in Figure 6. The restriction that compounds with the same charge may not react was kept. The energy thresholds were kept as in the previous explorations, and no other constraints were added besides the templates. The condensed data of the resulting networks are shown in rows 2, 4, and 5 of Table 1. If no templates are enforced (row 2), the exploration quickly reaches the point of combinatorial explosion. After 3 days of trial generation, the exploration was stopped at 25 million generated trials. An estimated 40 years of CPU time would have been spent working through these trials. However, we suspect that the final number of reaction trials would have been much higher than that.

**Figure 9:**
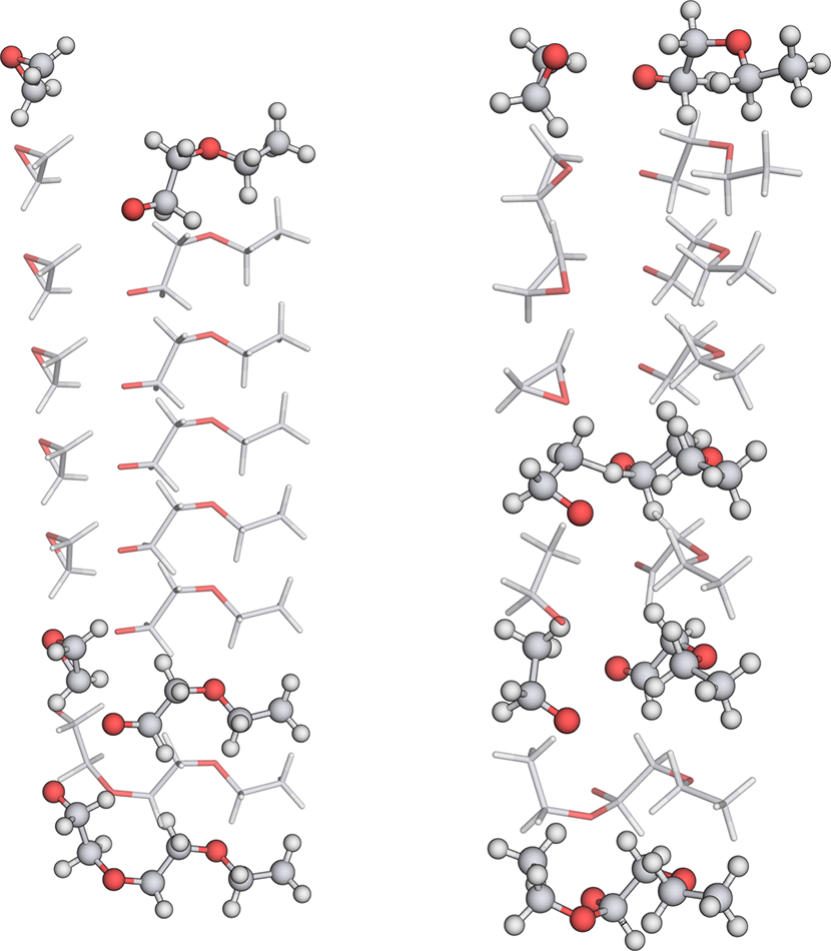
Two exemplary minimum energy paths of reactions which occur in the explored networks. On the left is the expected oligomerization of ethylene oxide, and on the right is an alternative reaction of the same starting materials that proceeds through an intermediate ethoxide anion. The structures shown with balls as atoms are (from top to bottom) starting material, transition state (intermediate ethoxide anion,) and product. The vertical position of structures indicates their relative position along the minimum energy path.

The explorations, constrained by the templates (row 4), converge with 6415 elementary step trials, reaching the *n* = 2 oligomer and generating 479 compounds, out of which 68 are deemed active compounds according to the thresholds set. It can be noted that the success ratio of the elementary step trials sunk now that the templates were not applied to the exact systems they were generated from. However, it is still higher than the initial exploration that generated the templates. The total runtime for this exploration was 72.5 CPU·h. Two elementary steps contained in the resulting reaction network are shown in Figure 9. As seen in row 5 of Table 1, increasing the molecular mass boundaries to the equivalent of *n* = 3 and exploring it further quickly lead to a combinatorial explosion, even with a restriction to templates. A better kinetic model would be required to cull further the combinatorial explosion in the more automated explorations aimed at higher molecular weights. The crude model applied here does allow for infinitely long chains of steps that are energetically uphill (Δ*G* > 0) as long as each increase is lower than 50 kJ/mol. A better kinetic model, such as an explicit microkinetic one, would determine the chains as infeasible or unlikely and disallow the exploration of compounds that result from them. Development toward the inclusion of such models^42^ in Chemoton is currently underway^9^ but had not been concluded at the time of preparation of this work.

**Figure 10:**
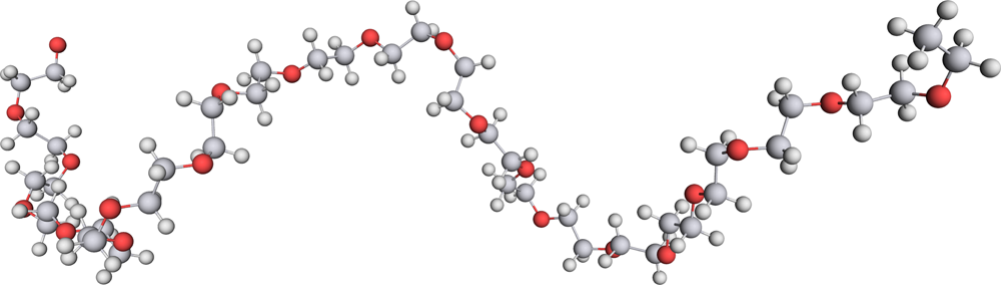
Single conformer of poly(ethylene oxide) with *n* = 20, as discovered with autonomous reaction exploration accelerated by filtering with a single reaction template.

However, reducing the number of applied templates to improve performance is also possible. The results of repeating the exploration with the template database existing of only one manually curated template are given in row 6 of Table 1. Within less than 1000 CPU hours, the exploration autonomously generated polymers up to *n* = 20. An exemplary structure of the compound grouping all conformers of the polymer with *n* = 20 is shown in Figure 10. The resulting network included only the starting material compounds and one compound for each polymer for *n* = 1 to *n* = 20. In this example, the runtime was dominated by the cost of each reaction trial, especially those of the larger polymers, rather than the number of trials. It highlights well that operator-introduced templates are a viable option to speed up and control explorations. However, simultaneously, it shows that with only 22 compounds, no side reactions are probed.

Because the templates shall also be transferable across reaction networks, additional explorations for polymerizing poly(propylene glycol) or poly(propylene oxide) (PPO) were carried out. The same filters and settings were applied here as in the previous examples. However, the reaction barrier threshold was increased to Δ*G*^‡^ < 95 kJ/mol, and an overestimated hydride abstraction reaction (see the following section) was removed from the network, meaning that the resulting products were listed as not active. The condensed results are presented in rows 7, 8, and 9 of Table 1.

For a molecular mass limit corresponding to *n* = 2 of the given polymer (row 8), the success rate of the reaction trials is slightly above 30%, matching that of the analogue PEO case. At the same time, the number of explored compounds is less than half of that in the PEO study (row 4). This is likely due to removing the single hydride abstraction reaction from the set of allowed templates. The runtimes align with those of the PEO example, considering the increased system size and the reduced number of trials.

In addition to the test with a database of 25 templates extracted from the brute force exploration, a network based on the single operator-generated template was also explored (row 9 of Table 1). This exploration was limited to *n* = 5. Again, the reaction trials’ success rate is very high. A significantly larger amount of compounds is reported in the PEO example with only one template and a limit of *n* = 20. This is because Molassembler and therefore Chemoton differentiate the two enantiomers resulting from the reaction of propylene oxide and that both an attack at the one and two positions of propylene oxide are explored. There would thus have been 3^5^ = 243 possible polymers up to *n* = 5. However, some of these possible polymers are not likely to be found due to the lack of conformational sampling and the resulting overestimation of some reaction barriers and subsequent deactivation of compounds.

### Network Analysis Based on Reaction Templates

While the reaction templates can steer and accelerate reaction network exploration, they are also a viable concept to help categorize, visualize, and understand the data within a reaction network.

Comparing more detailed results of the explorations of the PEO polymerization, we can query the reaction network and find that the initial exploration contains 221 unique reactions. In comparison, the re-exploration constrained by templates (Table 1, row 3) contains 17 unique reactions, and the network converged to a molecular mass of equivalent to *n* = 2 contains 1118 unique reactions (Table 1, row 4). It would now be possible to visually inspect and compare all of these reactions as they are stored in the database. However, this analysis is expensive (human time), error-prone, and possibly entirely unfeasible for more extensive networks, even if energy bounds are applied beforehand.

**Figure 11:**
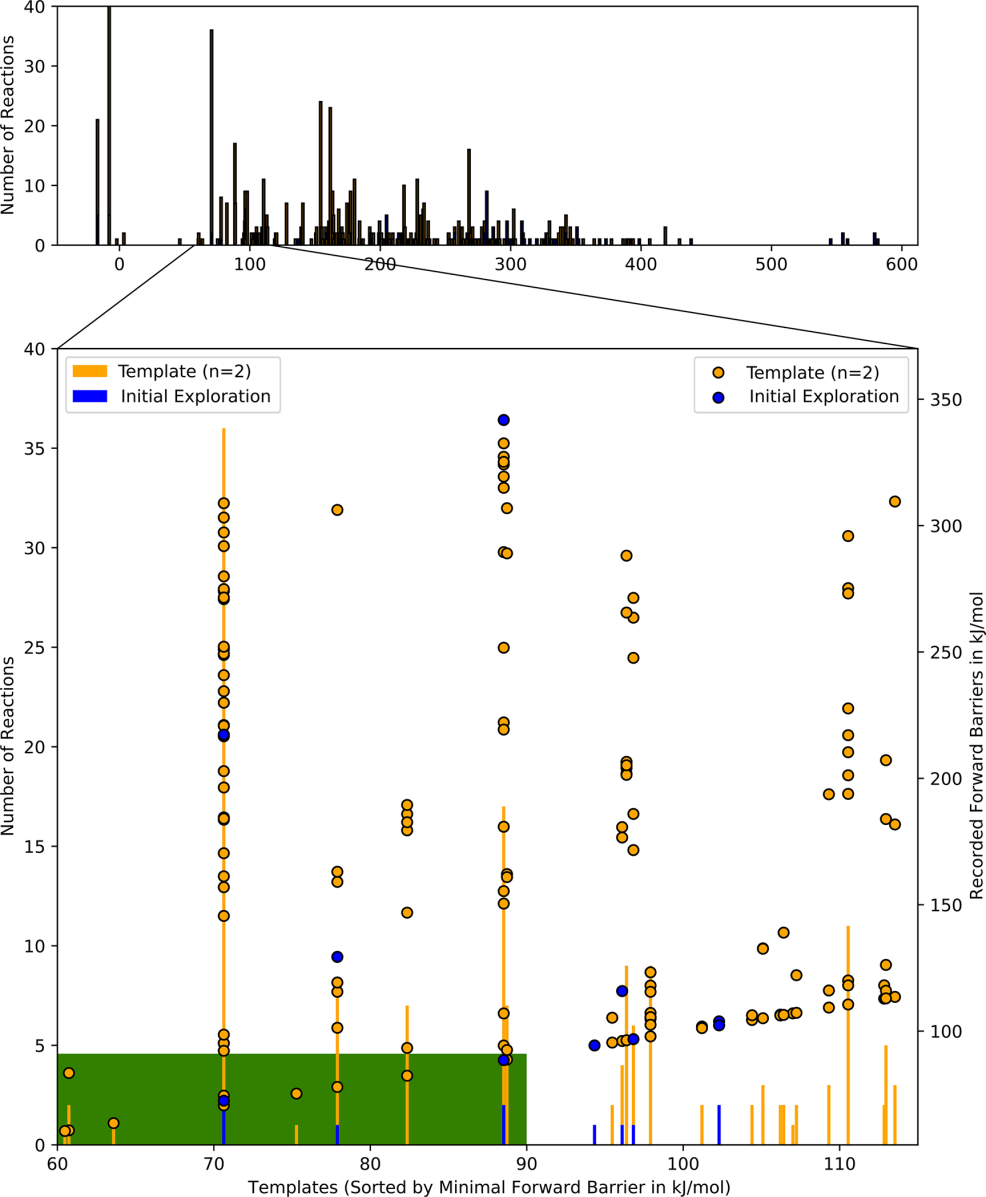
Templates occurrence in the generation of poly(ethylene oxide), sorted by their minimal forward reaction barrier. Each bar indicates one template, its height indicating the frequency of occurrence within the given reaction network. Points in the expanded view (bottom) indicate all recorded reaction barriers per template. The green area indicates chosen kinetic thresholds for template application (width) and product activation (height).

Here, the reaction templates allow a prior categorization. Grouping all reactions into templates, we find that all reactions in the re-exploration match those in the initial set of 79 templates. The mass-bound exploration generates 160 new templates. Thus, we can conclude that besides the intended template reactions, the NT2 algorithm generates some alternative reaction paths. For a closer look at the reactions, we can count the number of reactions per template and plot these as a bar plot, with each bar positioned according to the lowest recorded elementary step barrier per template. The plot is shown at the top of Figure 11 and is a spectrum unique to the given network. Analyzing the data shown in the top part of Figure 11, we can first note that four reaction templates (bars or peaks) with close to zero or negative reaction barriers are listed with a high occurrence. These reactions are hydride abstractions and hydride abstractions with concerted rearrangements. The chosen electronic structure method, GFN2-xTB (with implicit solvation), reports an affinity for free hydride ions in the solution. An example reaction of these templates is the reaction of ethanolate to acetaldehyde. The minimum energy path (electronic energy) reports a small barrier and a reaction energy of around −200 kJ/mol. Similarly, reactions that match the templates are reported as barrier-free when correcting electronic energies to Gibbs energies. While it would have been a viable option to switch methods to another semiempirical approximation, we believe it is more valuable to point out these results as they were obtained. We want to highlight that these oddities are instantly visible with the given presentation based on reaction templates. At the same time, they may have gone unnoticed when buried deep in an extensive reaction network that has to be analyzed by brute force visual inspection. That is not to say that no other automated checks could have also spotted these reactions. Given that the primary goal of this work is to investigate the network growth with respect to the reaction template choices, we continued to generate data with the chosen electronic structure method, despite these odd reactions. However, the products resulting from these reactions were set to be inactive in the PPO investigation, as discussed in that section.

Focusing on the expanded part (bottom) of Figure 11, we can note that many of the reaction templates group reactions with barriers (circles, second *y*-axis) on a range that spans more than 100 kJ/mol. A notable gap at around 150 kJ/mol can be seen, indicating that the recorded reactions for the highlighted templates generally fall into two categories. Those that are possibly reachable and relatively close to the minimal reported barrier, and those that are more unfavorable by more than 50 kJ/mol. A result of this analysis may be that in a more exhaustive exploration, a threshold for activating compounds of Δ*G*^‡^ < 150 kJ/mol may be a reasonable choice. Comparing the template occurrences of the initial exploration with those of the mass-converged one, we can observe that all of the initial templates that were applied (Δ*G*_min_^‡^ < 90 kJ/mol) are repeatedly occurring in the larger reaction network. This was to be expected. For two of the initial templates (at Δ*G*_min_^‡^ around 96–98 kJ/mol), we can see that the NT2 algorithm generated additional occurrences without the exact reaction coordinates being trialed (the templates are above the 90 kJ/mol threshold, indicated by the green block). Furthermore, the NT2 setup allowed for four groups of reactions to be found below the given energy threshold of (Δ*G*^‡^ < 80 kJ/mol), which would have been missing in the case of direct, double-ended elementary step explorations.

The highest bar in the expanded portion of Figure 11 (at about 72 kJ/mol) is the main reaction of the polymerization. Figure 9 on the left-hand side shows an example of such a reaction channel. It can be seen that the lowest initially recorded energy is undercut by one of the additional occurrences. Furthermore, it can be seen that many higher barriers are found. Inspecting the template for this reaction (see Figure 7a), we can see that this template also fits the reaction with an already polymerized ether. A closer inspection with SCINE Heron(43) confirms that the reported high energy barriers can be attributed to these. Figure 9 on the right-hand side shows an example of such a reaction channel.

## 5 Conclusions and Outlook

This work defines reaction templates based on existing molecular graph representations in SCINE Molassembler. The templates group reactions and encode all necessary information to generate elementary steps if matching reagents are supplied. The chosen template definition depends explicitly on the reacting atoms and their local environment.

The templates were tested on the polymerization of ethylene oxide and propylene oxide. It was demonstrated that templates are viable filters for reaction branches in chemical reaction networks. They decrease the overall number of reaction trials that are executed and additionally increase the success rate of reaction trials by more selectively neglecting unsuccessful ones. In future applications, pairing reaction templates with microkinetic modeling will allow for more routine explorations of reaction networks. Especially explorations with computationally more demanding electronic structure methods, such as those based on density functional theory, will benefit from this increased ease of automated steering.

Furthermore, we have briefly shown that reaction templates can be used to group and analyze data stored in reaction networks, making it more accessible to a human operator. The manual generation of reaction templates by interactive, real-time explorations will be part of a future release of SCINE Heron.^43^

Further investigations into additional abstractions based on atom types are possible extensions of the current template definition. Grouping atoms based on their position in the periodic table, i.e., grouping all halogen leaving groups, would be a step toward higher-level abstractions in chemical reaction networks akin to the concepts of reagents/synthons which are grouped by functionality rather than molecular graph identity. This extension is a goal we have mentioned previously.^4^ It would be particularly interesting for *a priori* analyses of possible reaction cascades in retrosynthetic planning.

This work focused on the templatization of occurring reactions and the propagation of reactive subgraphs of molecules. It stands to reason that it is equally beneficial to propagate knowledge about inert subgraphs of molecules. However, the knowledge of inertness is much more context-dependent than the knowledge of existing reactivity, and thus, it is more complex to store and propagate. Adding this second type of templatization would implicitly generate two bounds to the space of unknown reactivity and possibly allow for even more efficient searches of novel reactivity in chemical reaction networks.

## 6 Data Availability Statement

The reaction networks, summarized in Table 1, are publicly available on Zenodo.^44^ The data set contains the reaction networks referenced in rows 1, 4, 6, 8, and 9 of Table 1. All networks are stored in MongoDB databases. They are formatted according to the specifications encoded in the SCINEDatabase version 1.1.0.^45^ Those rows of Table 1 that are not individually part of the upload to Zenodo are subnetworks of those uploaded. The networks referenced in rows 3 and 7 are a subset of the uploaded networks referenced in rows 4 and 8, respectively. Due to size limitations, the incomplete networks for rows 2 and 5 were excluded from the upload. The given estimates for networks referenced in rows 2 and 5 can be generated by continued explorations based on the included networks of rows 1 and 4, respectively. All networks were stripped of the stored raw outputs for compression.

The template extraction and matching algorithms are part of a new Python3 package for automated reaction templates in SCINE Art. All data shown in this work was generated with a prerelease of version 1.0.0. The finalized release of SCINE Art version 1.0.0 is made available free and open-source (BSD 3-clause license), see refs (46) and (47). The underlying molecular graphs were generated with a prerelease of the SCINE package Molassembler version 2.0.0, which maintains the same license as version 1.2.1.^48^

## Supporting Information Available

Overview of key Chemoton settings for the generation of elementary step trials with the NT2 algorithm in the initial exploration and template application (PDF)
