# Supplementary material for: Accelerating Reaction Network Explorations with Automated Reaction Template Extraction and Application
Source: J Chem Inf Model. 2023 May 22;63(11):3392–403. doi: 10.1021/acs.jcim.3c00102 (PMC10268957; doi:10.1021/acs.jcim.3c00102)
Supplement: Supplementary file 1 — ci3c00102_si_001.pdf [file ci3c00102_si_001.pdf]

Supporting Information for: Accelerating Reaction Network Explorations with Automated Reaction Template Extraction and Application

Jan P. Unsleber[a,1]

[a] Laboratory of Physical Chemistry, ETH Zurich,
Vladimir-Prelog-Weg 2, 8093 Zurich, Switzerland
[1] jan.unsleber@phys.chem.ethz.ch; ORCID: 0000-0003-3465-5788

Detailed Exploration Settings

Table S1: Overview of key Chemoton settings for the generation of elementary step trials with the NT2 algorithm in the initial exploration and template application. (—: not applicable)

Molecularity	Setting	initial	template
Bimol.	Multiple attack directions per fragment	yes	yes
	# rotamers 1-on-1	2	2
	# rotamers 2-on-2	1	1
	Max. # bond formations total	2	—
	Max. # intermol. bond formations	2	—
	Max. # intramol. bond formations	1	—
	Max. # intramol. bond dissociations	1	—
	Multiple conformers per compound	no	no
Unimol.	Max. # bond modifications	2	—
	Max. # bond formations	1	—
	Max. # bond dissociations	1	—
	Multiple conformers per compound	no	no
